# Limited genomic diversity and convergent adaptation of *Brucella melitensis* isolated from human in East China, from 2011 to 2024

**DOI:** 10.3389/fmicb.2025.1663555

**Published:** 2025-10-10

**Authors:** Lan Huang, Lu Zhou, Nan Zhang, Weizhong Zhou, Buyun Xu, Jie Hong, Wei Zhang, Ying Zhang, Ke Xu, Changjun Bao, Hai Jiang, Zhongming Tan, Jingxin Li

**Affiliations:** ^1^School of Public Health, Southeast University, Nanjing, China; ^2^National Health Commission Key Laboratory of Enteric Pathogenic Microbiology, Jiangsu Provincial Center for Disease Control and Prevention, Nanjing, China; ^3^School of Public Health, National Vaccine Innovation Platform, Nanjing Medical University, Nanjing, China; ^4^College of Veterinary Medicine, Nanjing Agricultural University, Nanjing, China; ^5^Prenatal Diagnosis Center, Nanjing Drum Tower Hospital, The Affiliated Hospital of Nanjing University Medical School, Nanjing, China; ^6^National Key Laboratory of Intelligent Tracking and Forecasting for Infectious Diseases, National Institute for Communicable Disease Control and Prevention, Chinese Center for Disease Control and Prevention, Beijing, China

**Keywords:** human brucellosis, *Brucella melitensis*, core-genome MLST, pan-genome, core-genome SNP

## Abstract

**Objective:**

This study aimed to investigate the genomic epidemiology of *Brucella melitensis* in Jiangsu Province, a typical low-endemic region in East China where the incidence of human brucellosis has been increasing in recent years. Accordingly, a molecular epidemiological study was conducted on the 2,552 reported brucellosis patients in Jiangsu province, from 2011 to 2024.

**Methods:**

All *B. melitensis* isolated from these patients were sequenced using next-generation sequencing (NGS), and 515 strains met the criteria for subsequent analysis. Core genome multi-locus sequence typing (cgMLST), pan-genome analysis and core genome single nucleotide polymorphism (cgSNP) were utilized to analyze genomic characteristics and establish the epidemiological linkages among global strains.

**Results:**

Among 515 isolates, 439 (85.24%) and 505 (98.06%) were identified as *B. melitensis biovar 3* and sequence type 8(ST8), respectively. cgMLST further classified them into 28 core gene sequence types (cgSTs), including four novel genotypes (cgST1586-cgST1589) discovered in this study, whose identification expands the global cgMLST database and provides new markers for epidemiological surveillance. According to the cgSNP-based phylogenetic analysis, two distinct clades were persistently circulating within Jiangsu Province. One clade demonstrated significant genetic clustering with the Middle East strains, the other clade was closely linked to the hyper-endemic regions in China. Pan-genome analysis revealed their high homology, with core proteins primarily involved in amino acid transport and metabolism. Over the past 14 years, these isolates have exhibited limited genetic diversity and may be evolving toward a genotype that is better adapted to the host and environment.

**Conclusion:**

The human brucellosis in Jiangsu is mainly attributed to imported infections through various patterns, which is consistent with the typical epidemiology characteristics observed in low-endemic regions. The identification of four novel cgSTs and evidence of genomic evolutionary changes provide important insights to strengthen surveillance and guide targeted control strategies for brucellosis in East China.

## 1 Introduction

Brucellosis is a common zoonotic disease caused by the *Brucella genus*, affecting both animals and humans (Franco et al., 2007). The widespread prevalence of brucellosis has posed a serious global threat to the livestock industry and public health, with an estimated annual occurrence of 1.6–2.1 million new human cases (Laine et al., 2023). According to the reports, the disease is more prevalent in low-income areas, such as the Mediterranean region, Middle East and Asia, which have the highest incidence of brucellosis (Ali et al., 2024). Since the mid-1990s, the cases of human brucellosis in China have continued to increase and spread throughout the country (Li et al., 2013). Notably, even East China, a non-traditional epidemic area, has seen a rapid surge in the prevalence of brucellosis. As a province primarily focused on agriculture and manufacturing, Jiangsu has a relatively low rate of intensive sheep farming and is considered as a typical low-incidence region for brucellosis in East China (Ma et al., 2025; Cheng et al., 2023). However, its annual incidence has been reported higher than that of other provinces with comparable agricultural structures in recent years (Liu et al., 2025), a phenomenon that highlights the importance for conducting detailed epidemiological investigation.

*Brucella* is a gram-negative, facultative, intracellular pathogen that can infect a wide range of mammals. Humans are typically infected by direct contact with infected animals or contaminated materials (Qureshi et al., 2023). Currently, the *Brucella genus* contains 12 accepted species, of which the pathogen associated with human diseases includes *Brucella melitensis, Brucella abortus, Brucella suis and Brucella canis* (Zhao et al., 2021). Among these, *B. melitensis* is the predominant pathogen of human brucellosis in China (Tian et al., 2017) and is also responsible for the human infections in Jiangsu Province (Wang et al., 2025).

Various genetic tools have been employed to explore the evolution and geographic origins of the *Brucella genus*. Multi-locus sequence typing (MLST) (Whatmore et al., 2007) and multiple-locus variable number tandem repeat analysis (MLVA) (Le Fleche et al., 2006) are considered as traditional effective methods for investigating and tracing the source of *Brucella*. With the widespread adoption of next-generation sequencing technology (NGS), it has experienced significant advancements and is commonly used to distinguish *Brucella* strains now (Pelerito et al., 2020; Janowicz et al., 2018). Based on NGS with high genetic resolution, more precise analytical methods have been developed, such as core genome MLST (cgMLST), *in silico* MLVA, and core genome single nucleotide polymorphism (cgSNP) (Holzer et al., 2021; Brangsch et al., 2023). These methods provide more comprehensive traceability and deep insights for highly homologous *B. melitensis*.

In recent years, the pressure of brucellosis prevention and control in East China has been increasing (Liu et al., 2025). However, genomic epidemiological studies of *B. melitensis* remain limited, and the long-term epidemiological and evolutionary dynamics in this region are still poorly understood. Therefore, this study aims to elucidate the epidemiological characteristics of human brucellosis in Jiangsu Province from 2011 to 2024 and to explore the genetic diversity of the *B. melitensis* isolated from humans using a variety of the latest genetic methods. The findings are expected to provide a scientific foundation for optimizing prevention and control strategies, contributing to the limited body of genomic epidemiological research on *B. melitensis* in low-endemic regions.

## 2 Materials and methods

### 2.1 Epidemiological data collection

The epidemiological information of brucellosis patients was collected from Nationwide Notifiable Infectious Diseases Reporting Information System, including case numbers, age, gender, occupation, onset time and location. Demographic data were extracted from the Jiangsu Provincial Bureau of Statistics, and geographical information was acquired from the National Basic Geographic Information Center. R 4.3.3 software was used to present the epidemiological and bioinformatic analysis, and further refined for clarity and aesthetics with Adobe Illustrator 2025.

### 2.2 Strain isolation and identification

In accordance with the case definition of the Chinese Center for Disease Control and Prevention, the confirmed brucellosis patients must be accompanied by epidemiological exposure, clinical manifestations, and confirmatory diagnosis (standard agglutination test ≥ 100, Coombs IgG, complement fixation test, or isolation of *Brucella spp*) (Jiang et al., 2020). As the isolation and culture of organism remain the gold standard for diagnosis (Di Bonaventura et al., 2021), local medical institutions perform *brucella* isolation and culture for all suspected patients who present with epidemiological exposure and clinical manifestations. These isolates are then submitted for centralized preservation. For this study, the isolates were cultured at 37 °C for 48 h before further identification, and the species identification was conducted in compliance with the methodologies recommended by Guidelines for the Diagnosis of Human Brucellosis (WS 269–2019) in China (National Health Commission of the People's Republic of China., 2019). Specifically, genus-level confirmation was performed using BCSP31-PCR, and species-level identification using AMOS-PCR, with isolates yielding a 731 bp fragment in AMOS-PCR being definitively classified as *B. melitensis*.

### 2.3 Whole genome sequencing (WGS) and genotyping

All *B. melitensis* isolates were included for whole genome sequencing by MGISEQ-2000. The genomic nucleotide of these strains was extracted through the FastPure Bacteria DNA Isolation Mini Kit (Vazyme Biotechnology Co., Ltd. Nanjing, China), and quantified by Qubit 2.0 Fluorometer. The raw sequencing data were processed for quality filtering and assembly through CLC Genomics Workbench v23.0.1 software. Briefly, reads were quality-trimmed using the Trim Sequences tool (default settings) and assembled *de novo* with the *De Novo* Assembly tool, with short contigs (< 1,000 bp) removed to ensure reliability. All assemblies were confirmed again and retained for subsequent genomic analyses. Among the 2,552 brucellosis patients reported between 2011 and 2024, a total of 515 strains were successfully isolated from them and met the criteria for subsequent analysis.

Based on WGS, these isolates were subjected to MLST and MLVA genotyping to quantify genetic diversity. *In silico* MLVA analysis, 16 loci were used to characterize the strains through FastRCR 6.8 software (Kalendar et al., 2017), and dendrogram based on MLVA-16 profiles was constructed by BioNumerics5.0 software (Applied Maths, Belgium). Moreover, MLST-9, MLST-21 and cgMLST genotyping, along with application for new MLST genotypes were conducted using the PubMLST.org website (https://pubmlst.org/organisms/brucella-spp) (Jolley et al., 2018). BioNumerics5.0 software was also used to analyze the evolutionary relationships of allelic cgMLST by unweighted pair group method with arithmetic mean (UPGMA). Strains that exhibited close genetic relationships (allelic differences ≤ 5 allelic genes) were classified into a single cluster in the minimum spanning tree (MST), which is consistent with previously applied thresholds in *Brucella* genomic epidemiology studies (Schaeffer et al., 2021; Janowicz et al., 2018).

### 2.4 Phylogenetic analysis

The 160 representative strains in this study were selected based on epidemic information and the number of isolates with same core gene sequence type (Supplementary Table 1), given MLST analysis showed high homogeneity while cgMLST revealed distinct genotypic diversity. This selection strategy ensured that the chosen subset represented both the genomic diversity and the spatiotemporal distribution of the 515 isolates. The 77 global strains were curated from GenBank (https://www.ncbi.nlm.nih.gov/datasets/genome/?taxon=29459) according to their geographic isolation locations and sequencing data quality (Supplementary Table 2), resulting in a total of 237 strains subjected to phylogenetic analysis. Parsnp 2.0 was used to generate dendrogram based on cgSNP (Kille et al., 2024), and the SNP distances between strains were calculated through HarvestTools (Treangen et al., 2014). The tree was visualized and edited using iTOL (Interactive Tree of Life) v7.1.1 (Letunic and Bork, 2024). Epidemiological data, including onset time and geographic origin, were systematically integrated with the corresponding cgMLST and cgSNP clustering analyses to enhance the comprehensive understanding of the genomic and epidemiological relationships.

### 2.5 Gene annotation

Virulence factors (VF) genes, antimicrobial resistance factors (AMR) genes and prophage genes of the *B. melitensis* genome were identified using the Virulence Factors of Pathogenic Bacteria Database (VFDB, update to August 14, 2024) (Liu et al., 2022), the Comprehensive Antibiotic Resistance Database (CARD 3.2.6) (Alcock et al., 2023) and PHASTEST (Wishart et al., 2023), respectively. Among them, the identification of VF and AMR genes was performed by batch alignment in CLC Genomics Workbench v23.0.1 software using default parameters, only hits meeting >90% sequence identity and >90% coverage thresholds were considered valid.

### 2.6 Pan-genome analysis

All *B. melitensis* isolates in this study and 785 *B. melitensis* isolates retrieved from GenBank were subjected to pan-genome analysis. The genome data were processed by Prokka 1.14.3 to annotate and collect files in General feature format (GFF) (Seemann, 2014). Roary 3.13.0 was employed to extract the pangenome (sum of all genes found across strains), core genome (genes present among all strains), shell genome (genes present among more than one strain, but not in all), and strain-specific genes (genes present only in one strain) (Page et al., 2015). EggNOG-mapper 2.1.10 was utilized for functional annotation and providing COG (clusters of orthologous groups) categories of all the gene-related proteins (Cantalapiedra et al., 2021). Unless otherwise specified, all analyses were conducted using the default parameters provided by the respective software packages. The pan-genome curve was modeled using Heaps' law *y* = *AX*^*B*^+*C*, in which B > 0 indicates an open pangenome. The core-genome curve was modeled by exponential regression model (*y* = *Ae*^*Bx*^+*C*) (Tettelin et al., 2008; Yang et al., 2016).

## 3 Result

### 3.1 Epidemiological characteristics

**Figure 1:**
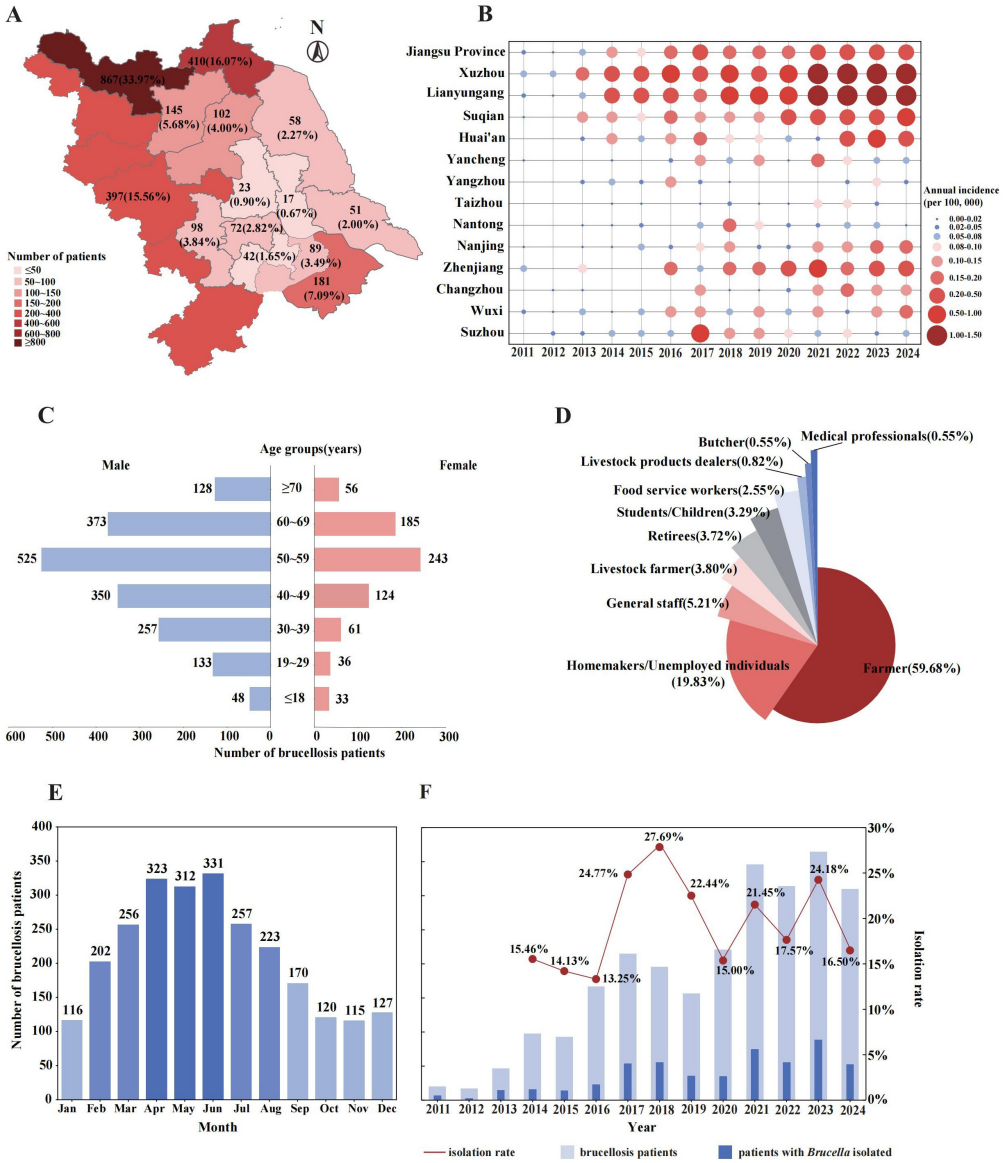
Epidemiological characteristics of reported brucellosis patients in Jiangsu Province and neighboring cities in Anhui Province, from 2011 to 2024. **(A)** The spatial distribution of brucellosis patients accumulated over 14 years, numbers indicate the total patients reported in each city. **(B)** The annual incidence of brucellosis in Jiangsu Province and its cities, shown by bubble size and color intensity. **(C)** The demographic characteristics of all reported brucellosis patients. **(D)** Occupational distribution of brucellosis patients. **(E)** Seasonal distribution of brucellosis patients by month. **(F)** Annual number of brucellosis patients and the corresponding isolation rate of *Brucella* strains between 2011 and 2024.

From 2011 to 2024, a total of 2,552 brucellosis patients were reported in Jiangsu Province, located in East China. Among them, 2,155 (84.44%) were permanent residents of Jiangsu, 397(15.56%) were residents of neighboring Anhui province who sought medical treatment in Jiangsu (Figure 1A). During the 14-year period, the average annual incidence rate in Jiangsu Province was 0.18 per 100,000 individuals. The incidence peaked at 0.34 per 100,000 in 2023 and has remained above 0.03 per 100,000 since 2021. The annual number of reported patients displayed a fluctuating upward trend (Figure 1F), with notable increase observed in 2016 and 2021. Significant spatial distribution differences were observed across cities in Jiangsu (Figure 1A and Supplementary Figure 1D), exhibiting a general pattern of higher incidence in the northern areas, a relatively lower rate in the southern areas, and the lowest incidence in the central areas. Central areas in Jiangsu, including Taizhou City, Yangzhou City, and Nantong City, exhibited consistently lower annual incidence rates (Figure 1B). In contrast, southern areas such as Suzhou City, Wuxi City, Changzhou City, Zhenjiang City, and Nanjing City were more susceptible to sporadic clustering events, with Suzhou recording the highest incidence rate (0.54 per 100,000) in 2017. Northern areas, including Yancheng City, Huai'an City, Suqian City, Lianyungang City, and Xuzhou City, demonstrated a persistent upward trend in annual incidence rates. Since 2014, Lianyungang and Xuzhou have alternated as the city with the highest annual brucellosis incidence in Jiangsu (with the exception of 2017), with Xuzhou reporting significantly more brucellosis patients than other cities. Additionally, the number of patients among Anhui residents increased steadily over time, peaking at 70 patients in 2023, accounting for 19.23% of the total reported cases that year (Supplementary Figure 1C).

Among all reported brucellosis patients, the number of male patients was significantly higher than that of female, with a male-to-female ratio of 2.46:1. Patients were distributed across all age groups, ranging from 3 months to 86 years, with a mean age of 50.3 ± 15.3 years. As shown in Figure 1C, the age group of 50–59 years had the most patients (768, 30.09%) regardless of gender. Additionally, the middle-aged and elderly individuals aged 40–69 were the main affected population (*n* = 1,800), accounting for 70.53% of the total reported patients. The occupational distribution (Figure 1D) revealed that farmers (1,523, 59.68%) were the highest-risk occupation for brucellosis in East China, followed by homemakers/unemployed individuals (19.83%, 506). Additionally, common related occupations such as livestock farmers (97, 3.80%), livestock product dealers (21, 0.82%), and butchers (14, 0.55%) were also observed.

The onset time of reported patients showed a distinct seasonal distribution (Figure 1E), with the peak period occurring from April to June in Jiangsu Province and its surrounding regions. The number of patients generally decreased from October to January of the following year. Among the 2,552 reported brucellosis patients, *brucella* strains were successfully isolated from 515 individuals, resulting in an average isolation rate of 20.18%. The highest isolation rate (54, 27.69%) was observed in 2018, while the lowest (22, 13.25%) was recorded in 2016, and the isolation rates stabilized within the range of 15 to 20% between 2019 and 2024 (Figure 1F).

### 3.2 Identification and MLST

A total of 515 strains isolated from brucellosis patients were identified as *B. melitensis*, and *B. melitensis b3* was the predominant serotype (439, 85.24%), followed by *B. melitensis b2* (66, 12.82%) and *B. melitensis b1* (10, 1.94%). In the results of MLST, three genotypes were identified by MLST-9 (ST8, ST39 and ST121) and four were identified by MLST-21 (ST8, ST82, ST168 and ST169). The MLST-9 and MLST-21 of these strains showed high consistency, with ST8 being the most common genotype (505, 98.06%), and only one strain was identified as ST39 and ST82, respectively (Table 1). Additionally, two new MLST-21 sequence type were discovered in this study and officially named as ST168 and ST169, whose cgSTs profile are both cgST1132. The primary distinctions between them and ST8 were observed in the *prpE* and *gyrB* loci.

**Table 1:** Distribution of sequence types (STs) of *B. melitensis* isolates based on MLST-9 and MLST-21.

**MLST-9**	**Number of strains (%)**	**MLST-21**	**Number of strains (%)**
ST8	507 (98.45%)	ST8	505 (98.06%)
		ST169	2 (0.39%)
ST121	7 (1.36%)	ST168	7 (1.36%)
ST39	1 (0.19%)	ST82	1 (0.19%)

**Figure 2:**
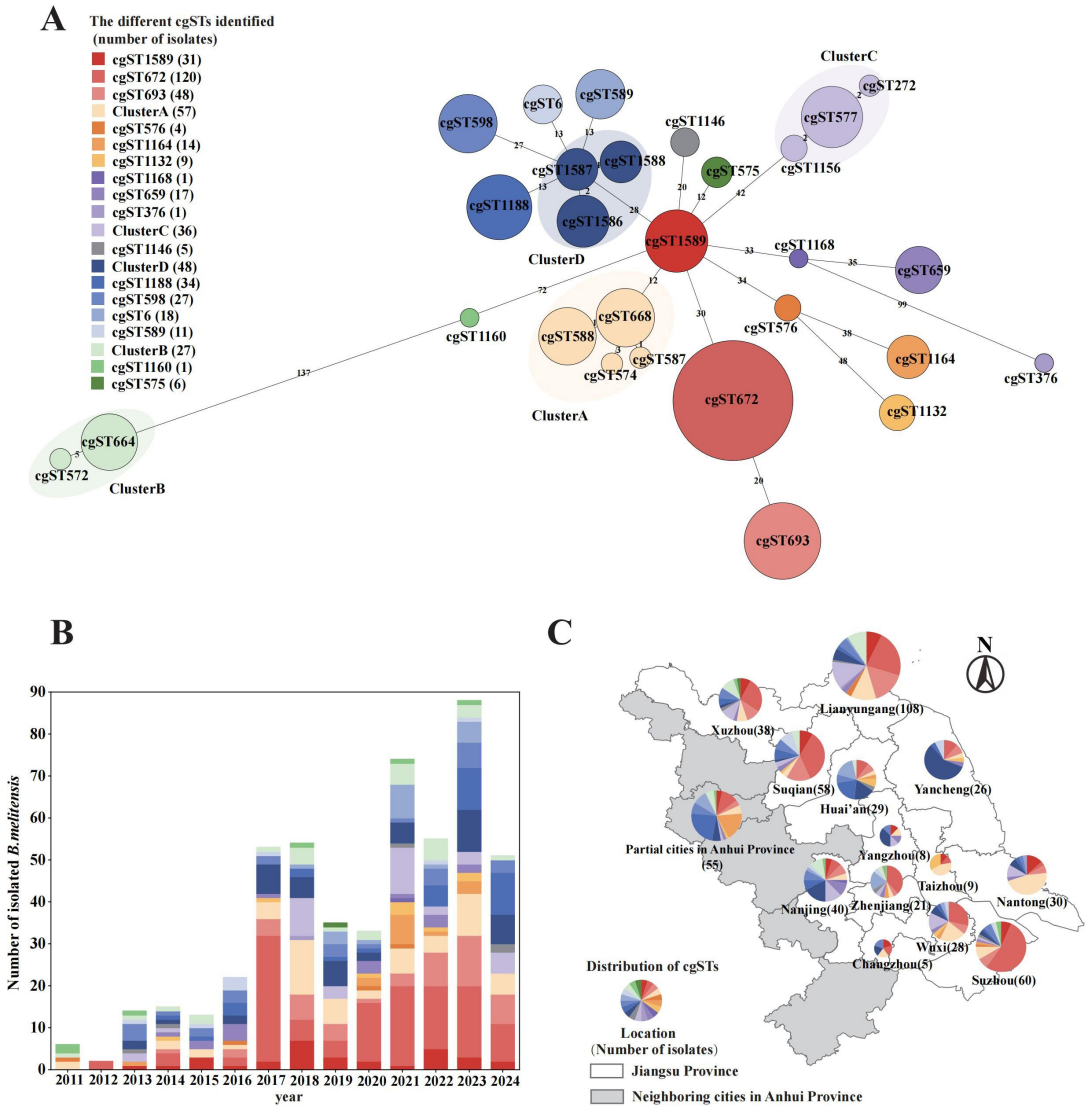
Evolutionary relationships and spatiotemporal distributions of 28 core genome sequence types (cgSTs) identified from 515 *B. melitensis* isolates. **(A)** Minimum spanning tree based on allelic cgMLST differences of 515 strains. **(B)** Temporal distribution of cgSTs by year. **(C)** Spatial distribution of cgSTs in Jiangsu Province and several neighboring cities in Anhui Province. All three panels share the same color legend for cgSTs.

Further analysis performed by the cgMLST database revealed that 28 core gene sequence types were identified, and subsequently grouped into 21 clusters based on genetic distance (Figure 2A). With the increasing number of isolates collected each year, the genetic diversity also expanded, indicating the continuous introduction of foreign genotypes (Figure 2B). Among these, cgST672 (120, 23.30%) was the most prevalent genotype, which could be consistently isolated across all study years. Additionally, four novel genotypes were discovered in this study that were not present in the existing database. These newly identified genotypes were subsequently submitted to the global database and designated as cgST1589, cgST1588, cgST1587, and cgST1586. Strains identified as cgST1589 (31, 6.02%) were located near the center of the minimum spanning tree and exhibited a distinct association with other strains. In Jiangsu Province, the cgST1589 strains were initially isolated in 2013 and have been continuously observed in subsequent years. Despite being grouped into Cluster D because of their close genetic distance, cgST1586 (21, 4.08%), cgST1587 (13, 2.52%) and cgST1588 (13, 2.52%) exhibited epidemiological heterogeneity in their geographic distribution. Specifically, the cgST1586 strains were predominantly found in northern Jiangsu, cgST1587 strains were mainly in southern region, and cgST1588 strains were concentrated in Yancheng City. The overall spatial distribution (Figure 2C) indicated southern Jiangsu was dominated by cgST672 strain (52/154, 33.77%), while the northern region exhibited a more diverse range of genotypes. Cluster B strains (including cgST664 and cgST572), which exhibit a relatively greater allelic distance from other strains, were widely distributed throughout different geographical and temporal range of Jiangsu.

### 3.3 Phylogenetic analysis

**Figure 3:**
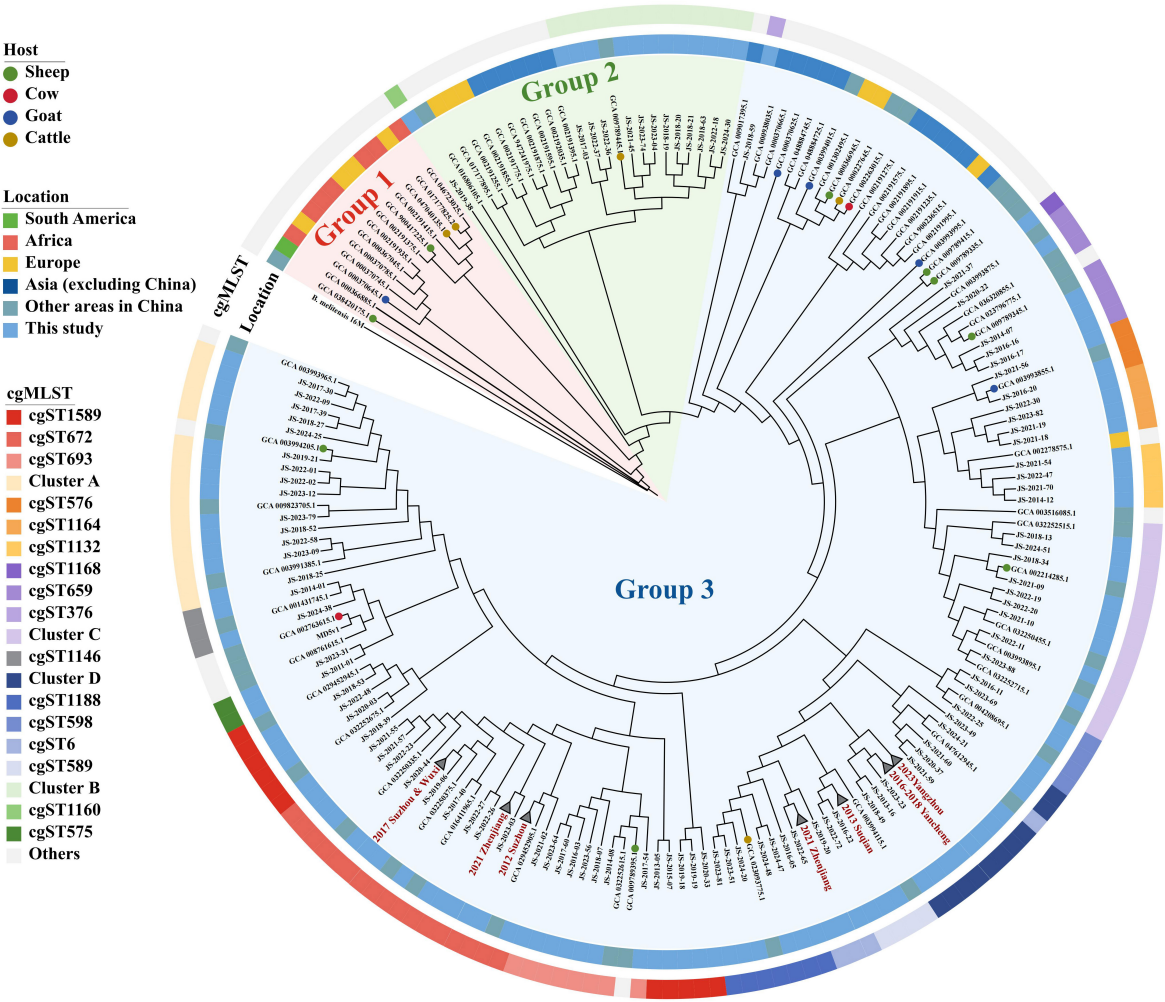
Phylogenetic trees of 237 *B. melitensis* strains, collapse branches indicate previously identified outbreaks. The inner circle indicates the region where the strains were isolated. The outer circle represents the cgST of all strains, and the color legend is consistent with Figure 2.

Global core genome SNP phylogenetic analysis revealed that the 237 strains were clustered into three groups (Figure 3, Supplementary Figure 2, Supplementary Table 2). Group 1 consisted of thirteen strains, primarily originating from Africa, Europe, and South America, as well as one strain isolated from the sheep in Inner Mongolia, China. Group 2 comprised twenty-four strains, including all cgST1160 and Cluster B strains identified in this study, along with strains from Europe and other Asian countries. Group 3 included the majority of strains in this study, with strains sharing the same cgST predominantly clustered together. Within this Group, apart from the cgST376 and cgST1132 strains, all other strains of this study exhibited genetic similarity exclusively to previously reported domestic strains. The cgST376 strain, corresponding to unique ST39 (MLST-9) and ST82 (MLST-21), exhibited the highest similarity to strains from Malaysia and Thailand. Additionally, the cgST1132 strains were closely related to a strain isolated from Russia, and their MLST-21were all novel ST168 or ST169.

**Figure 4:**
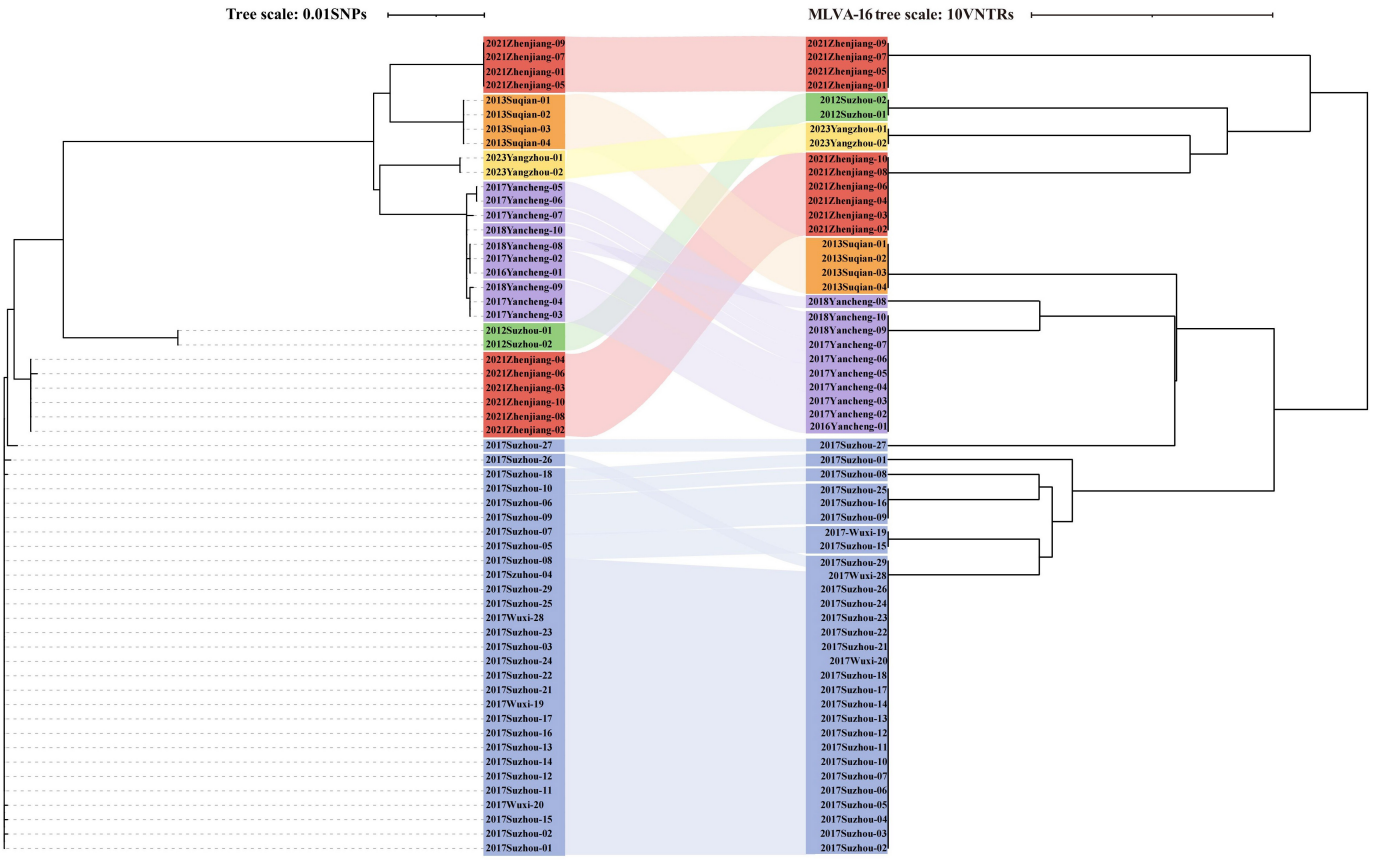
The relationship between cgSNP and MLVA-16 profiles in six identified outbreaks (*n* = 57). Dendrogram based on cgSNP is presented on the **left**, dendrogram based on MLVA-16 profiles is displayed on the **right**, and the corresponding outbreak is compared in the middle using a Sankey plot for visualization.

Furthermore, all six outbreaks confirmed by epidemiological investigation were in Group3, and the pairwise SNP distances of the isolates in the same outbreak were ≤ 3 SNPs. Except for 2021 Zhenjiang outbreak, each outbreak was characterized by a single cgST. A comparative analysis of the clustering concordance between cgSNP and MLVA-16 in these outbreaks demonstrated that both methods could effectively cluster outbreak events. However, the clustering concordance of cgSNP was more pronounced, as it could generally group isolates from the same outbreak into a single branch despite internal variations within an event (Supplementary Table 3, Figure 4).

### 3.4 Virulence factors, antimicrobial resistance factors and prophages

Based on the VFDB database, a total of 66 virulence factor genes were identified in 515 *B. melitensis*, with no significant differences observed among these strains. These genes are mainly related to LPS (31, 46.97%), the Type IV secretion system (14, 21.21%), and VirB type IV secretion system (12, 18.18%), which are responsible for immune modulation, effector delivery system, regulatory system and intracellular survival (Table 2). The sole identified antimicrobial resistance factor is the Brucella suis *mpr*F, present in all isolates, which confers resistance to peptide antibiotics by altering the antibiotic target. Additionally, Dinoroseobacter phage vB_DshS-R5C was identified as the unique and shared prophage across isolates.

**Table 2:** Virulence factors identified in 515 *B. melitensis* strains.

**Virulence factors**	**Function**	**Genes**
Lipopolysaccharide (LPS)	Immune modulation, invasion and intracellular survival	*gmd, per, wzm, wzt, wbkB, wbkC, wbpL, acpXL, manBcore, manCcore, lpxK, waaA/kdtA, wboA, wbdA, htrB, lpxE, pgm, kdsB, pmm, manAoAg, manCoAg, wbpZ, lpsA, wbkA, lpxD, fabZ, lpxA, lpxB, lpxC, kdsA, lpsB/lpcC*
Type IV secretion system (T4SS) secreted effectors	Effector delivery system	*bspA, bspB, bspC, bspE, bspF, bspL, vceA, vceC, BPE043, BPE005, BPE275, BPE123, ricA, sepA*
VirB type IV secretion system	Effector delivery system	*virB1, virB2, virB3, virB4, virB5, virB6, virB7, virB8, virB9, virB10, virB11, virB12*
Adherence	Adhesion and invasion	*bigA, bigB, bmaB/OmaA, bmaC*
TIR domain containing protein	Immune modulation and evasion	*btpA, btpB*
Sensory-regulatory adaptation system (BvrS/BvrR)	Regulatory systems	*bvrS, bvrR*
Cyclic beta 1–2 glucan synthetase (CβG)	Intracellular survival	*cgs*

### 3.5 Pan-genome analysis

**Figure 5:**
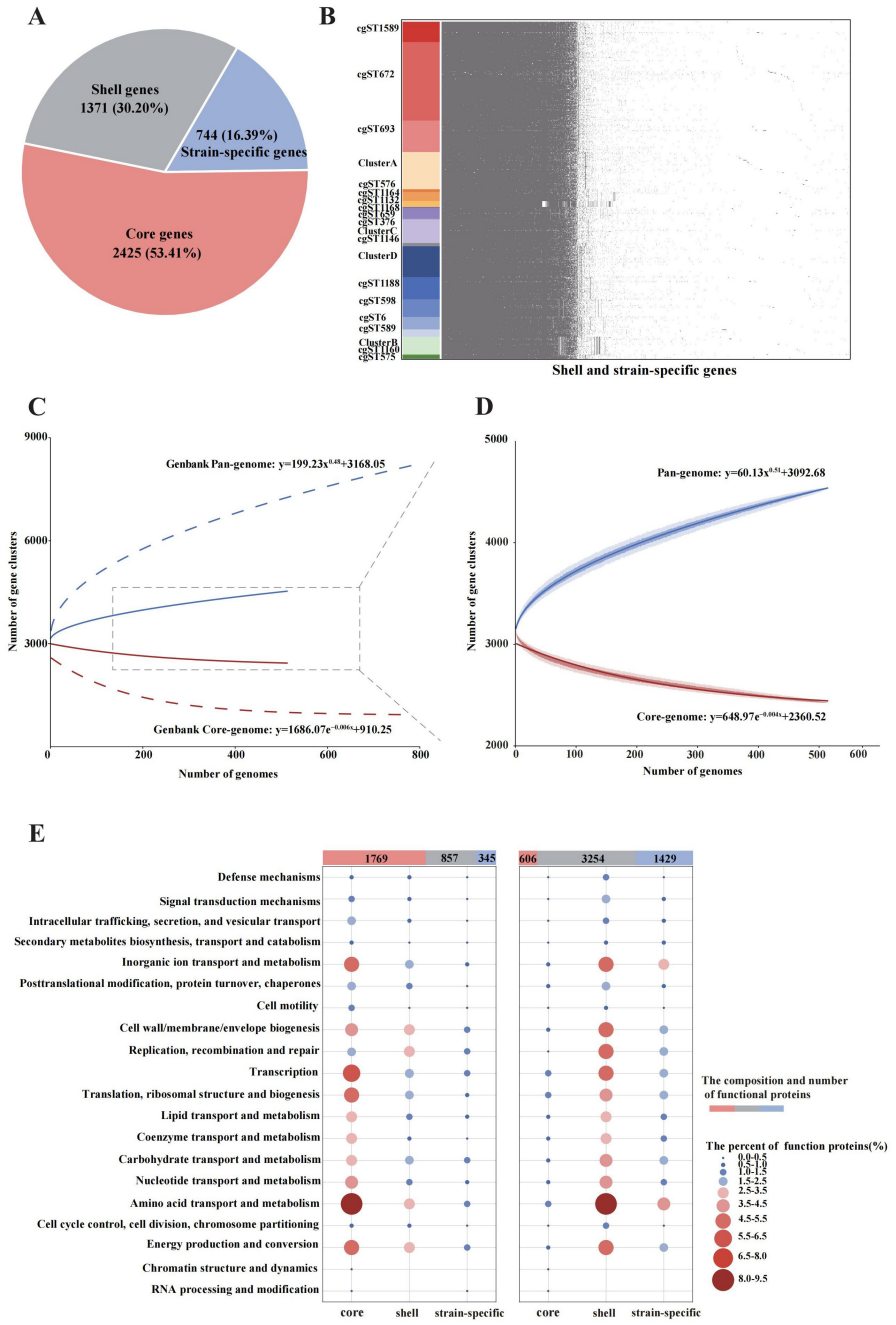
Pan-genome analysis of 515 *B. melitensis* from this study and 785 strains from GenBank. **(A)** Composition of the pan-genome in the 515 isolates. **(B)** The presence and absence matrix of the shell genes and strain-specific genes in 515 strains. **(C)** The accumulation curves of the pan-genome and core-genome based on both the 515 isolates and 785 strains from Genbank. **(D)** The accumulation curves of the pan-genome and core-genome based only on the 515 isolates. **(E)** The distribution and COG functional characteristics of the gene families on the 515 isolates (left) and 785 Genbank strains (right).

In the pan-genome analysis of 515 *B. melitensis* strains, a total of 4,540 gene families were identified, including 2,425 core gene families, 1,371 shell gene families, and 744 strain-specific genes (Figure 5A). Minimal differences were observed in shell genes and strain-specific genes across different cgMLST strains, except for cgST1132, cgST1160 and Cluster B (Figure 5B). With the inclusion of genomes, the pan-genome expanded continuously in accordance with Heaps' law (Figure 5D, *y* = 60.13*x*^0.51^+3092.68). In contrast, the core genome exhibited an inverse decreasing trend (*y* = 648.97*e*^−0.004*x*^+2360.52) as more genomes were added. Compared with the pan-genome analysis of 785 *B. melitensis* strains from GenBank, the strains in this study displayed higher homology in both the pan-genome curve and the core-genome curve (Figure 5C). In the COGs analysis of the 515 strains, 1,769 core-gene-associated functional proteins (core proteins), 857 shell proteins and 345 strain-specific proteins were successfully annotated. The main core proteins (1,769, 59.54%) were primarily associated with amino acid transport and metabolism, inorganic ion transport and metabolism, and transcription (Figure 5E). Conversely, the COGs analysis of 785 GenBank strains showed that shell proteins were the most abundant (3,254, 61.52%) functional proteins, and also primarily related to amino acid transport and metabolism, inorganic ion transport and metabolism, and energy production and conversion.

## 4 Discussion

Located in the eastern plain of China, Jiangsu Province has traditionally been considered as a low-endemic region for brucellosis. However, the number of Jiangsu patients has been increasing over the past 14 years, and the annual incidence of brucellosis has risen to 0.03 per 100,000 population since 2021. Concurrently, a sustained rise in imported patients from neighboring Anhui Province has been observed, even reaching 19.23% of all reported cases in 2023. Genotypic analysis further demonstrated substantial overlap in cgST profiles between these imported isolates and strains circulating in Jiangsu residents, suggesting that imported patients may have contributed to the recent increase in incidence. In addition, pronounced regional heterogeneity was observed across Jiangsu Province, with incidence rates highest in northern areas, followed by southern and central areas (*P* < 0.001). For instance, the significantly higher prevalence in Xuzhou City and Lianyungang City compared to other cities, aligns with their frequent mutton trade links to hyper-endemic northern provinces and longstanding dietary preferences for small ruminant products (Zhang et al., 2023). This indicates that we should not only focus on infections caused by livestock trade but also on imported patients arising from population movement. Similar to other low-endemic regions (Tan et al., 2023), farmers aged 40–69 are the most vulnerable population in Jiangsu, as they are directly exposed to infected animals. However, individuals in non-traditional occupations are also at a high risk of infection due to consumption of or direct contacting with contaminated food (Bao et al., 2022). Consequently, the occupational spectrum has broadened, the age distribution has expanded, and transmission routes have become more complex, thereby posing substantial challenges for prevention and control. In response to the population characteristics and various infection patterns of brucellosis patients in Jiangsu Province, it is essential to enhance cooperation with the agricultural, forestry and veterinary departments to achieve effective control from the source. These epidemiological findings in Jiangsu Province are consistent with those reported across East China, where provinces share broadly similar demographic characteristics and transmission patterns (Zhao et al., 2025; Shi et al., 2024). As a typical low-endemic province in this region, Jiangsu can reasonably serve as a representative area to illustrate the regional epidemiological and genomic features of human brucellosis. Accordingly, our study further explored the genomic epidemiology of *B. melitensis* to provide insights relevant to low-endemic areas in East China.

In this study, the serotype and genotype of 515 *B. melitensis* were highly homologous. Specifically, *B. melitensis biovar 3* and ST8 are the predominant types in Jiangsu Province and across China (An et al., 2021; Li et al., 2025; Wang et al., 2020), and ST8 has likewise been frequently reported in neighboring countries (Ayoub et al., 2025; Akar and Erganis, 2022). Moreover, the newly named ST168 and ST169 shared the same cgMLST (cgST1132), which were officially named in 2024 and no related publications have been reported to date. However, global phylogenetic analysis revealed that these isolates were closely related to an isolate from Russia in 1983, whose MLST-21 has been reclassified as ST169. This connection highlights the significance of traditional methods in bridging contemporary findings with legacy databases. Similar to the epidemiological characteristics, cgMLST analysis showed that cgST672 was the dominant genotype in southern Jiangsu. In contrast, the greater genetic diversity observed in northern regions was likely attributed to the complexity of transmission patterns. Importantly, cgST672 strains were also dominant in the 2017 Suzhou outbreak, one of a large-scale spread across cities and years in Jiangsu Province. Fortunately, no colonization was detected in the subsequent analysis, as the SNP distance between the cgST672 strains isolated later in Suzhou and the outbreak strains was significantly large (SNP > 10). Although no cgST672 *B. melitensis* strains have been reported in published articles, two *B. melitensis* strains isolated from Qinghai Province have been documented in PubMLST database (Jolley et al., 2018). As the predominant genotype responsible for outbreaks in Jiangsu Province, cgST672 strains also appear to be prevalent in hyper-endemic regions, highlighting the necessity for nationwide attention. Due to the identification of four novel genotypes in this study, several Chinese strains retrieved from GenBank were successfully reclassified as cgST1589 (Supplementary Table 1), which were initially isolated in Inner Mongolia in 2014. These associations highlight the potential correlation between *B. melitensis* in Jiangsu and those from domestic hyper-endemic regions from a genetic point of view. Notably, the closer a node is to the center, the greater the cgST number tends to be, and the smaller the genetic distance typically becomes. Consequently, this phenomenon may be attributed to advancements in WGS technology, or it could be inferred that *B. melitensis* is progressively evolving toward a common favorable genotype to better adapt to its host and environment.

According to the cgSNP analysis, the strains in this study were divided into two main clades, exhibiting distinct evolutionary relationships. One clade includes cgST1160, cgST664 and cgST572 strains, which belong to Group2 in global phylogenetic analysis. The cgST1160 strain showed close genetic similarity to one strain isolated from Qinghai Province. Previous reports have reported genetic links between strains from Qinghai to those from Shandong and Anhui provinces (Zhao et al., 2021; Xue et al., 2023), both of which border Jiangsu Province, thereby deeply elucidating the genetic association among strains in the East China and Qinghai. The cgST664 and cgST572 strains typically related to the strains from Middle Eastern countries and neighboring European countries, which exhibit the highest seroprevalence levels in ruminants compared to other endemic regions (Musallam et al., 2016). As important countries along the Silk Road, these countries previously engaged in frequent trade with China mainly through ruminants, which may have facilitated the widespread spread of *B. melitensis* (Liu et al., 2024, 2020). Taking Iran as an example, recent genomic and MLVA analyses of *B. melitensis* from both humans and livestock consistently identified the Eastern Mediterranean lineage, with ST8 as the predominant genotype (Dadar and Alamian, 2025; Dadar et al., 2023). This pattern aligns with our findings and indirectly supports the hypothesis that transmission is closely associated with the movement of ruminants. The other clade contained most strains and was mainly related to strains from other provinces in China, such as Inner Mongolia, Heilongjiang, Xinjiang, Liaoning, and Hainan. This indicates that Jiangsu is continually imported by introductions from northern endemic regions, which remain the primary domestic sources of human infection. Moreover, the coexistence of these two clades highlights a dual epidemiological risk, with sporadic international incursions coinciding with persistent domestic transmissions from hyper-endemic northern provinces. These findings emphasize the importance of incorporating genomic surveillance into routine risk assessment and strengthening inter-provincial control measures in East China.

As a traceability tool for outbreak investigation, cgSNP showed better clustering concordance in the comparative analysis of 57 strains. Its main advantage lies in the high resolution that enables precise discrimination of isolates, but the absence of a unified standard for the SNP of *B. melitensis* remains an unignorable limitation. In contrast, MLVA-16 is relatively variable, it is quick and has been widely used (Ferreira et al., 2012). Therefore, in phylogenetic analysis, MLVA-16 is well-suited for real-time outbreak investigations, while SNP analysis is better suited for retrospective traceability studies. The combined use of both methods in this study took advantage of their complementary strengths, improving the timeliness of outbreak surveillance and maintaining high discriminatory resolution for *B. melitensis*.

High-resolution cgMLST and cgSNP analyses revealed that the 515 *B. melitensis* strains differed mainly in internal genotypes. Furthermore, pan-genome analysis revealed a substantial number of core gene families, including those associated with virulence factors, antimicrobial resistance, and prophage identified in this study, thereby further confirming the high degree of genetic homogeneity among these strains. Functional annotation of these core genes is primarily associated with amino acid transport and metabolism, a metabolic characteristic that has been consistently observed in pan-genome studies of *Brucella spp*. (Mazwi et al., 2024; Yang et al., 2024) and aligns with established biological fact (Ronneau et al., 2016). In contrast, the 785 publicly available genomes span multiple centuries and continents, and their shell genes account for the majority of inter-strain variability. Nevertheless, consistent with this study, functionally annotated proteins of these shell genes were also enriched in amino acid transport and metabolism. Therefore, genes related to amino acid transport and metabolism constitute a core functional pathway in the highly conserved *B. melitensis*, supporting intracellular survival, long-term persistence, and adaptation to diverse host environments, thereby underscoring their fundamental role in pathogen biology.

The limited genetic diversity observed among the isolates aligns with previous findings that *B. melitensis* exhibits a high degree of genomic homogeneity (Ksibi et al., 2025), which is indicative of purifying selection linked to its intracellular lifestyle. Existing studies have further suggested that such genetic stability facilitates convergent adaptation, whereby distinct lineages evolve toward similar metabolic and virulence-related traits that enhance persistence within the host environment (Machelart et al., 2020). This evolutionary constraint may help explain the relatively conserved clinical manifestations and transmission pathways of human brucellosis, while also underscoring highlight key implications for vaccine development.

This study employed a range of molecular epidemiological tools to characterize the genetic features of *B. melitensis* in Jiangsu Province, but there are limitations that should not be ignored. The strains in this study were all isolated from brucellosis patients, and no strains were obtained from indigenous ruminants. The absence of animal isolates limits the application of a One Health perspective. Integrating parallel sampling of ruminants during future outbreak surveillance could provide a more comprehensive understanding of cross-species transmission and contribute to the development of more targeted control strategies. In addition, this study was unable to directly correlate genetic variations with clinical severity or individual transmission, and analyses were therefore limited to overall transmission patterns. Future surveillance efforts should integrate such data to facilitate more precise genomic–epidemiological correlations.

## 5 Conclusion

The epidemiological analysis conducted in this study revealed that Jiangsu Province, a region with low endemicity, has been significantly influenced by various types of imported infections. Molecular epidemiology revealed that the *B. melitensis* isolated from brucellosis patients were primarily classified into two clades, which have been circulating for the past 14 years. Furthermore, multiple genetic characterization studies have indicated that these isolates exhibit limited diversity and may be evolving progressively to better adapt to the host and environment. Considering the current situation of brucellosis in this typical low-endemic area of East China, prevention and control should prioritize the growing threat of imported infections through reinforced livestock quarantine and inter-provincial traceability systems to reduce the risk of local transmission. Although imported cases predominate, the possibility of indigenous infections cannot be excluded, underscoring the need for sustained surveillance of local livestock and continuous genomic monitoring to detect and interrupt potential transmission chains. Taken together, these measures provide tailored strategies for strengthening brucellosis prevention and control in low-endemic settings such as Jiangsu Province.

## Data Availability

The datasets presented in this study can be found in online repositories. The data regarding four newly identified genotypes are available in the National Center for Biotechnology Information (NCBI) under BioProject PRJNA1248685. The other datasets are available in the National Microbiology Data Center repository (nmdc.cn), accession number NMDC10019898.

## Supplementary material

Supplementary Figure 1 Geographic characteristics of human brucellosis patients in Jiangsu Province, 2011-2024. **(A)** Location of Jiangsu Province and Anhui Province in East China. **(B)** Three geographical regions of Jiangsu Province. **(C)** Annual number of reported brucellosis patients in Jiangsu Province from 2011 to 2024, stratified by household registration. **(D)** Cumulative incidence of brucellosis in northern, central, and southern Jiangsu, with dots representing values for individual cities. Statistical analysis using Bonferroni-corrected pairwise χ^2^ tests indicated significant differences between all three regions (*P* < 0.001).

Supplementary Figure 2 Phylogenetic trees of 237 *B. melitensis* strains, and branch length are not be ignored.

Supplementary Table 1 The basic information and genotyping of 160 strains for phylogenetic analysis, in this study.

Supplementary Table 2 The basic information and genotyping of 77 strains for phylogenetic analysis, from Genbank.

Supplementary Table 3 The MLVA genotypes of 57 strains related to outbreak cases, in this study.

Supplementary Table 4 Primers used for PCR and in silico MLVA-16 analysis.

## References

[B1] Akar K. Erganis O. (2022). Evaluation of the genetic profiles of strain from Turkey using multilocus variable number tandem repeat analysis (MLVA) and multilocus sequence typing (MLST) techniques. Vet. Microbiol. 269:109423. 10.1016/j.vetmic.2022.109423 35462118

[B2] Alcock B. P. Huynh W. Chalil R. Smith K. W. Raphenya A. R. Wlodarski M. A.. (2023). CARD 2023: expanded curation, support for machine learning, and resistome prediction at the comprehensive antibiotic resistance database. Nucleic Acids Res. 51, D690–D699. 10.1093/nar/gkac920 36263822 PMC9825576

[B3] Ali S. Mushtaq A. Hassan L. Syed M. A. Foster J. T. Dadar M. (2024). Molecular epidemiology of brucellosis in Asia: insights from genotyping analyses. Vet. Res. Commun. 48, 3533–3550. 10.1007/s11259-024-10519-5 39230771

[B4] An C. H. Liu Z. G. Nie S. M. Sun Y. X. Fan S. P. Luo B. Y.. (2021). Changes in the epidemiological characteristics of human brucellosis in Shaanxi Province from 2008 to 2020. Sci. Rep. 11:17367. 10.1038/s41598-021-96774-x 34462483 PMC8405659

[B5] Ayoub H. Kumar M. S. Mehta R. Sethuraj S. E. Thomas P. Dhanze H.. (2025). Genomic insights into in India: stability of ST8 and the role of virulence genes in regional adaptations. Microbiol. Spectr. 13:e0264724. 10.1128/spectrum.02647-24 40272150 PMC12131837

[B6] Bao L. Cui J. R. Chen L. L. (2022). Epidemiological investigation of a human brucellosis outbreak in main urban area of Suzhou City from 2016 to 2017. Chin. J. Zoonoses. 38, 548–552. 10.46234/ccdcw2021.030 34595016 PMC8393115

[B7] Brangsch H. Sandalakis V. Babetsa M. Boukouvala E. Ntoula A. Makridaki E.. (2023). Genotype diversity of brucellosis agents isolated from humans and animals in Greece based on whole-genome sequencing. BMC Infect. Dis. 23:529. 10.1186/s12879-023-08518-z 37580676 PMC10426126

[B8] Cantalapiedra C. P. Hernandez-Plaza A. Letunic I. Bork P. Huerta-Cepas J. (2021). eggNOG-mapper v2: functional annotation, orthology assignments, and domain prediction at the metagenomic scale. Mol. Biol. Evol. 38, 5825–5829. 10.1093/molbev/msab293 34597405 PMC8662613

[B9] Cheng M. Quan J. Yin J. Liu X. Yuan Z. Ma L. (2023). High-resolution maps of intensive and extensive livestock production in China. Resour. Environ. Sustainability 12:100104. 10.1016/j.resenv.2022.100104

[B10] Dadar M. Alamian S. (2025). In silico MLVA Analysis of brucella melitensis from human and livestock in Iran. Curr. Microbiol. 82:74. 10.1007/s00284-024-03940-1 39777506

[B11] Dadar M. Brangsch H. Alamian S. Neubauer H. Wareth G. (2023). Whole-genome sequencing for genetic diversity analysis of Iranian *Brucella* spp. isolated from humans and livestock. One *Health* 16:100483. 10.1016/j.onehlt.2023.100483 36632477 PMC9827381

[B12] Di Bonaventura G. Angeletti S. Ianni A. Petitti T. Gherardi G. (2021). Microbiological laboratory diagnosis of human brucellosis: an overview. Pathogens 10:1623. 10.3390/pathogens10121623 34959578 PMC8709366

[B13] Ferreira A. C. Chambel L. Tenreiro T. Cardoso R. Flor L. Dias I. T.. (2012). MLVA16 typing of Portuguese human and animal Brucella melitensis and Brucella abortus isolates. PLoS One 7:e42514. 10.1371/journal.pone.0042514 22905141 PMC3419166

[B14] Franco M. P. Mulder M. Gilman R. H. Smits H. L. (2007). Human brucellosis. Lancet Infect. Dis. 7, 775–786. 10.1016/S1473-3099(07)70286-4 18045560

[B15] Holzer K. El-Diasty M. Wareth G. Abdel-Hamid N. H. Hamdy M. E. R. Moustafa S. A.. (2021). Tracking the distribution of *Brucella abortus* in Egypt based on core genome SNP analysis and in silico MLVA-16. Microorganisms 9:1942. 10.3390/microorganisms9091942 34576838 PMC8469952

[B16] Janowicz A. De Massis F, Ancora, M. Camma C. Patavino C. Battisti A.. (2018). Core genome multilocus sequence typing and single nucleotide polymorphism analysis in the epidemiology of *Brucella melitensis* infections. J. Clin. Microbiol. 56, e00517–18. 10.1128/JCM.00517-18 29925641 PMC6113479

[B17] Jiang H. Feng L. Lu J. (2020). Updated guidelines for the diagnosis of human brucellosis—China, 2019. China CDC Wkly. 2, 487–489. 10.46234/ccdcw2020.129 34594685 PMC8393123

[B18] Jolley K. A. Bray J. E. Maiden M. C. J. (2018). Open-access bacterial population genomics: BIGSdb software, the PubMLST.org website and their applications. Wellcome Open Res. 3:124. 10.12688/wellcomeopenres.14826.1 30345391 PMC6192448

[B19] Kalendar R. Khassenov B. Ramankulov Y. Samuilova O. Ivanov K. I. (2017). FastPCR: an in silico tool for fast primer and probe design and advanced sequence analysis. Genomics 109, 312–319. 10.1016/j.ygeno.2017.05.005 28502701

[B20] Kille B. Nute M. G. Huang V. Kim E. Phillippy A. M. Treangen T. J. (2024). Parsnp 2.0: scalable core-genome alignment for massive microbial datasets. Bioinformatics 40:btae311. 10.1093/bioinformatics/btae311 38724243 PMC11128092

[B21] Ksibi B. Smaoui F. Ben Ayed N. Guetat M. Mezghani S. Ktari S.. (2025). Genomic analysis of Brucella melitensis isolates recovered from humans in south Tunisia over 35 years between 1988 and 2022. BMC Microbiol. 25:98. 10.1186/s12866-025-03802-1 40011821 PMC11866833

[B22] Laine C. G. Johnson V. E. Scott H. M. Arenas-Gamboa A. M. (2023). Global estimate of human brucellosis incidence. Emerg. Infect. Dis. 29, 1789–1797. 10.3201/eid2909.230052 37610167 PMC10461652

[B23] Le Fleche P. Jacques I. Grayon M. Al Dahouk S. Bouchon P. Denoeud F.. (2006). Evaluation and selection of tandem repeat loci for a Brucella MLVA typing assay. BMC Microbiol. 6:9. 10.1186/1471-2180-6-9 16469109 PMC1513380

[B24] Letunic I. Bork P. (2024). Interactive Tree of Life (iTOL) v6: recent updates to the phylogenetic tree display and annotation tool. Nucleic Acids Res. 52, W78–W82. 10.1093/nar/gkae268 38613393 PMC11223838

[B25] Li W. Zeng L. Yuan R. Qi T. Liao H. Cao Y.. (2025). Genetic diversity atlas of *Brucella melitensis* strains from Sichuan Province, China. BMC Microbiol. 25:21. 10.1186/s12866-024-03739-x 39810148 PMC11731553

[B26] Li Y. J. Li X. L Liang S. Fang L. Q. Cao W. C. (2013). Epidemiological features and risk factors associated with the spatial and temporal distribution of human brucellosis in China. BMC Infect. Dis. 13:547. 10.1186/1471-2334-13-547 24238301 PMC3834885

[B27] Liu B. Zheng D. Zhou S. Chen L. Yang J. (2022). VFDB 2022: a general classification scheme for bacterial virulence factors. Nucleic Acids Res. 50, D912–D917. 10.1093/nar/gkab1107 34850947 PMC8728188

[B28] Liu G. Ma X. Zhang R. Lu J. Zhou P. Liu B.. (2024). Epidemiological changes and molecular characteristics of Brucella strains in Ningxia, China. Front. Microbiol. 15:1320845. 10.3389/fmicb.2024.1320845 38314436 PMC10835715

[B29] Liu Z. Shi Y. Xue C. Yuan M. Li Z. Zheng C. (2025). Epidemiological and spatiotemporal clustering analysis of human brucellosis—China, 2019–2023. China CDC Wkly. 7, 130–136. 10.46234/ccdcw2025.020 39931445 PMC11807248

[B30] Liu Z. Wang C. Wei K. Zhao Z. Wang M. Li D.. (2020). Investigation of genetic relatedness of brucella strains in countries along the silk road. Front. Vet. Sci. 7:539444. 10.3389/fvets.2020.539444 33490123 PMC7817895

[B31] Ma X. Liu Z. Geng Y. Zhao Y. Meng H. Chen M.. (2025). Changing trends in human brucellosis in pastoral and agricultural China, 2004–2019: a Joinpoint regression analysis. BMC Infect. Dis. 25:160. 10.1186/s12879-025-10561-x 39901073 PMC11792675

[B32] Machelart A. Willemart K. Zuniga-Ripa A. Godard T. Plovier H. Wittmann C.. (2020). Convergent evolution of zoonotic Brucella species toward the selective use of the pentose phosphate pathway. Proc. Natl. Acad. Sci. U. S. A. 117, 26374–26381. 10.1073/pnas.2008939117 33020286 PMC7584911

[B33] Mazwi K. D. Lekota K. E. Glover B. A. Kolo F. B. Hassim A. Rossouw J.. (2024). Whole genome sequence analysis of *Brucella* spp. from human, livestock, and wildlife in South Africa. J Microbiol. 62, 759–773. 10.1007/s12275-024-00155-8 39037482 PMC11436471

[B34] Musallam I. I. Abo-Shehada M. N. Hegazy Y. M. Holt H. R. Guitian F. J. (2016). Systematic review of brucellosis in the Middle East: disease frequency in ruminants and humans and risk factors for human infection. Epidemiol. Infect. 144, 671–85. 10.1017/S0950268815002575 26508323

[B35] National Health Commission of the People's Republic of China. (2019). Diagnosis for Brucellosis (WS 269–*2019)*. Beijing: National Health Commission.

[B36] Page A. J. Cummins C. A. Hunt M. Wong V. K. Reuter S. Holden M. T. G.. (2015). Roary: rapid large-scale prokaryote pan genome analysis. Bioinformatics 31, 3691–3693. 10.1093/bioinformatics/btv421 26198102 PMC4817141

[B37] Pelerito A. Nunes A. Núncio M. S. Gomes J. P. (2020). Genome-scale approach to study the genetic relatedness among strains. PLoS ONE 15:e0229863. 10.1371/journal.pone.0229863 32150564 PMC7062273

[B38] Qureshi K. A. Parvez A. Fahmy N. A. Abdel Hady B. H. Kumar S. Ganguly A.. (2023). Brucellosis: epidemiology, pathogenesis, diagnosis and treatment-a comprehensive review. Ann. Med. 55:2295398. 10.1080/07853890.2023.2295398 38165919 PMC10769134

[B39] Ronneau S. Moussa S. Barbier T. Conde-Alvarez R. Zuniga-Ripa A. Moriyon I.. (2016). Brucella, nitrogen and virulence. Crit. Rev. Microbiol. 42, 507–525. 10.3109/1040841X.2014.962480 25471320

[B40] Schaeffer J. Revilla-Fernandez S. Hofer E. Posch R. Stoeger A. Leth C.. (2021). Tracking the origin of Austrian human brucellosis cases using whole genome sequencing. Front. Med. 8:635547. 10.3389/fmed.2021.635547 33718408 PMC7943447

[B41] Seemann T. (2014). Prokka: rapid prokaryotic genome annotation. Bioinformatics 30, 2068–2069. 10.1093/bioinformatics/btu153 24642063

[B42] Shi X. G. Liu Y. Yang Y. Zhang R. Ren J. P. Guo S.. (2024). Epidemiological characteristics and spatial clustering of human brucellosis in Zhejiang province, 2018–2022. Dis. Surveillance 39, 1147–1150.

[B43] Tan Q. Wang Y. Liu Y. Tao Z. Yu C. Huang Y.. (2023). Molecular epidemiological characteristics of Brucella in Guizhou Province, China, from 2009 to 2021. Front. Microbiol. 14:1188469. 10.3389/fmicb.2023.1188469 37426016 PMC10326899

[B44] Tettelin H. Riley D. Cattuto C. Medini D. (2008). Comparative genomics: the bacterial pan-genome. Curr. Opin. Microbiol. 11, 472–477. 10.1016/j.mib.2008.09.006 19086349

[B45] Tian G. Z. Cui B. Y. Piao D. R. Zhao H. Y. Li L. Y. Liu X.. (2017). Multi-locus variable-number tandem repeat analysis of Chinese Brucella strains isolated from 1953 to 2013. Infect. Dis. Poverty 6:89. 10.1186/s40249-017-0296-0 28460642 PMC5412030

[B46] Treangen T. J. Ondov B. D. Koren S. Phillippy A. M. (2014). The Harvest suite for rapid core-genome alignment and visualization of thousands of intraspecific microbial genomes. Genome Biol. 15:524. 10.1186/s13059-014-0524-x 25410596 PMC4262987

[B47] Wang H. Xu W. M. Zhu K. J. Zhu S. J. Zhang H. F. Wang J.. (2020). Molecular investigation of infection sources and transmission chains of brucellosis in Zhejiang, China. Emerg. Microbes Infect. 9, 889–899. 10.1080/22221751.2020.1754137 32284015 PMC7241503

[B48] Wang W. Lu Z. Teng G. Yan Z. Huang L. Tan Z.. (2025). Phylogenetic evidence for nationwide expansion *Brucella melitensis* lineages drives the re-emerging and epidemic of human brucellosis in Jiangsu, China. Front. Cell. Infect. Microbiol. 15:1603234. 10.3389/fcimb.2025.1603234 40964059 PMC12436464

[B49] Whatmore A. M. Perrett L. L. Macmillan A. P. (2007). Characterisation of the genetic diversity of Brucella by multilocus sequencing. BMC Microbiol. 7:34. 10.1186/1471-2180-7-34 17448232 PMC1877810

[B50] Wishart D. S. Han S. Saha S. Oler E. Peters H. Grant J. R.. (2023). PHASTEST: faster than PHASTER, better than PHAST. Nucleic Acids Res. 51, W443-W450. 10.1093/nar/gkad382 37194694 PMC10320120

[B51] Xue H. Zhao Z. Wang J. Ma L. Li J. Yang X.. (2023). Native circulating *Brucella melitensis* lineages causing a brucellosis epidemic in Qinghai, China. Front. Microbiol. 14:1233686. 10.3389/fmicb.2023.1233686 37799605 PMC10547896

[B52] Yang X. Li Y. Zang J. Li Y. Bie P. Lu Y.. (2016). Analysis of pan-genome to identify the core genes and essential genes of Brucella spp. Mol. Genet. Genomics 291, 905–912. 10.1007/s00438-015-1154-z 26724943

[B53] Yang Z. L. Chai Z. L. Wang X. Zhang Z. H. Zhang F. W. Kang F. Q.. (2024). Comparative genomic analysis provides insights into the genetic diversity and pathogenicity of the genus. Front. Microbiol. 15:1389859. 10.3389/fmicb.2024.1389859 38721599 PMC11076708

[B54] Zhang N. Fang X. Y. Zhou W. Z. Tan Z. M. Liang S. Y. Wang X. C.. (2023). Epidemiological characteristics and temporal-spatial clustering analysis on human brucellosis in Jiangsu Province, 2006–2021. Sci. Rep. 13:20024. 10.1038/s41598-023-46690-z 37973934 PMC10654521

[B55] Zhao R. Sun R. Zhang F. (2025). Epidemiological characteristics and spatial clustering analysis of human brucellosis in Zibo City, Shandong Province, China, 2006–2024. Front. Public Health 13:1580265. 10.3389/fpubh.2025.1580265 40655220 PMC12245763

[B56] Zhao Z. J. Li J. Q. Ma L. Xue H. M. Yang X. X. Zhao Y. B.. (2021). Molecular characteristics of Brucella melitensis isolates from humans in Qinghai Province, China. Infect. Dis. Poverty 10:42. 10.1186/s40249-021-00829-0 33771234 PMC8004457

